# Extramedullary acute myeloid leukemia (eAML): Retrospective single center cohort study on clinico-pathological, molecular analysis and survival outcomes

**DOI:** 10.1016/j.amsu.2021.102894

**Published:** 2021-10-22

**Authors:** Khalid Halahleh, Yazan Alhalaseh, Dalia Al-Rimawi, Waleed Da'na, Kamal Alrabi, Nazmi Kamal, Isra Muradi, Hikmat Abdel-Razeq

**Affiliations:** a Internal Medicine Department, King Hussein Cancer Center, Amman, Jordan; b Research Office, King Hussein Cancer, Amman, Jordan; c Pathology Department, Hematopathology Section, King Hussein Cancer Center, Amman, Jordan; d Internal Medicine Department, University of Tripoli, Tripoli, Libya

**Keywords:** AML, Leukemia, Myeloid sarcoma, Extramedullary disease

## Abstract

**Introduction:**

extramedullary acute myeloid leukemia (eAML) is characterized by extramedullary tumor formation infiltrated by myeloid blasts, with or without maturation and effaced architecture. The clinical, genetic and molecular aspects and overall outcomes are well defined worldwide, but not well characterized in our region.

**Purpose and methods:**

This is a retrospective single center cohort study on 32 patients, who were identified over 10 years to study the clinical, pathologic and genetic-molecular aspects, and survival outcomes.

**Results:**

eAML is rare (1%), occurs at a younger age with male predominance. Central nervous system (CNS) with facial bone invasion is most commonly identified (34.4%). 45.5% were positive for conventional myeloid markers (MPO), CD33, CD117, and 36% positive for CD34 and CD68. 54% with normal karyotype had deleterious mutations on further testing. NGS revealed pathogenic mutations in 76%(N-9/17) and none tested positive for P53, IDH1 or IDH2. At a median follow up time of 43mo (range, 8.6–80mo); 37.5%(N-12) were in complete remission, 62.5%(N-20) relapsed. 28% of relapses were after allotransplant. 31%(N-10) alive and continued in complete remission(CR), and 69%(N-22) of patients have died.

Median overall survival (OS) is 18.4 and relapse free survival (RFS) 18.7 months. OS and RFS were significantly better in patients, who attained CR after induction (IC 11.9 mo vs zero; P = 0.0001; IC 12mo vs zero; P = 0.0001) compared to patients with relapsed disease; and in patients who received allo-transplant consolidation with median OS and RFS 42 vs 8.5mo (P = 0.002) and 42months vs 10 mo (P = 0.006). Thus allotransplant may be considered for all eligible patients in first CR.

**Conclusion:**

achievement of complete remission after induction therapy is associated with improved outcomes in eAML. Allotransplant in first complete remission may be the most effective modality for achieving long-term remissions.

## Highlights

• eAML is a rare entity and occurs at a younger in Jordan.
• It requires advanced methods for the diagnosis, and identification of targetable mutations, that may dictate therapeutic approaches.
• AML-like induction chemotherapy regimens remain the standard of care, and consolidation therapy is controversial.
• Failure to achieve complete remission is associated with worse survival outcomes.
• Allogeneic hematopoietic transplant may be considered in first complete remission.
• Post-transplant relapses are common and responsible for increased mortality and shorter survival.

## 1 Introduction

The diagnosis of AML is based on well-known WHO diagnostic criteria. Extramedullary site of disease can be the sole criteria, which can occur with or without bone marrow involvement [1,2]. Extamedullary AML(eAML) is characterized by a tumor mass infiltrated by myeloid blasts, with or without maturation and effaced architecture [[3], [4], [5]]. eAML can occur in the context of intramedullary AML, but may also occur in an isolated form without antecedent bone marrow involvement (isolated eAML), which is usually followed by marrow involvement during the course of the disease in 80–90% of cases, whereas the reminder occurs in the setting of other hematopoietic disorders including myelodysplastic syndrome, MDS, myeloproliferative neoplasms(MPN) or chronic myelomonocytic leukemia(CMML) and chronic myelogenous leukemia(CML) [[4], [5], [6]].

The true incidence of eAML is difficult to calculate, due to descriptive nature of studies without pathological confirmation. The rates of eAML with or without marrow involvement at diagnosis ranged between 0.2 to 2.8% and 0.6–0.8% and, reached 8% on autopsy studies among patients dying from AML [2,6,7]. Following allotransplant, the incidence has been reported to be 16–24% in total [[8], [9], [10]], 8–18% isolated and 2–8% with marrow involvement; which accounts for 7–46% of post-transplant relapses. eAML may occur in every organ, but most commonly involved sites are soft tissue/connective tissue (31–35%) and skin (11–46%), gastrointestinal system (10–19%). Reproductive organs (1–10%), bone (5–16%), head and neck (6–14%) and brain (4–11%) are less frequently encountered [5,[11], [12], [13], [14]].

eAML can easily misdiagnosed in 40% of cases especially with lymphomas. Histological confirmation is essential for diagnosis, as well as cytogenetic and molecular testing, when isolated forms is encountered in particular [6]. Imaging modalities including CT scans, MRI are of great value, but FDG-PET/CT appears to be the best modality for MS diagnosis [15]. There is no accepted standard of care in the treatment of eAML patients. Treatment modalities are influenced by several factors, including conventional prognostic and predictive markers for AML, such as age, chromosomal abnormalities and the presence of somatic mutations beside other aspects of being de novo or relapsed disease, isolated or with concurrent marrow involvement, prior allotransplant or not. Treatment results stems from retrospective studies and case series [[4], [5], [6], [7],^16^]. Local therapies including surgical resection and involved field radiotherapy should be considered for all patients with isolated forms [6,17]. Systemic therapy is reasonable, as 80–90% of cases will progress to AML within 4–6 months. Though, there is no specific regimen for eAML, but most patients were treated with anthracycline-cytarabine combinations [6]. 15–27% of patients who underwent allotransplant had intramedullary disease. The reported 5-year OS after allotransplant for patients with eAML ranges from 47 to 53% [16].

Herein, we report on 32 patients to analyze clinical, histological, genetic and molecular characteristics, treatment outcomes in patients with pathologically confirmed eAML.

## 2 Patients and methods

*This is a retrospective single center cohort study from a public Cancer Center*-King Hussein Cancer Center(KHCC). We screened Hematology-Bone Marrow Transplantation Program-Cancer Registry database of KHCC between January 2003 to September 2019, for patients with the diagnosis of AML, MDS, CMML, CML, MPN, with or without eAML. All eAML cases diagnosed based on WHO criteria were included [18]. Institutional review board (IRB) approval was obtained. Patients who had blasts in the CSF were labelled as extramedullary leukemia, and patients with liver and splenic involvement were not clubbed with eAML. 35 patients fulfilled the inclusion criteria. 3 patients were excluded as they were treated in another facility. Patient-disease-and treatment-related demographics were retrospectively collected from electronic patient's charts, including age, gender, blood counts, AML-FAB subtype, anatomic site, history of hematopoietic or other neoplastic disorders, histologic, genetic and molecular characteristics, immunphenotype using flow cytometry or immunohistochemistry, status of bone marrow disease at diagnosis, treatments given and long-term outcomes. The study has been reported in line with the STROCSS criteria [18], and Research Registry UIN: NCT05057299. www.clinicaltrials.com.

## 3 Diagnosis

The diagnosis was made based on clinical, radiological, histopathological, and cytogenetic-molecular evaluation, when possible. Bone marrow examination was done for all patients. Central nervous system (CNS) eAML, were diagnosed by contrast enhanced CT or MRI head in cases with clinical central nervous system symptoms. PET-CT was not done for most patients, based on physician's decision. Immunphenotype was determined, when possible. karyotyping, and FISH studies using specific probes for specific mutations and next generation sequencing (NGS) was done when possible, using 52 myeloid panel, and FLT-3 mutation by RT-PCR or NGS results were collected (supplement of NGS panel).

### 3.1 Pathology, immune-histochemistry and flow-cytometry

Pathology was reviewed by an expert hematopathologist, along with peripheral blood and bone marrow aspirate smears to confirm the diagnosis. The phenotypical profile was determined in 34% of cases. Myeloid markers were positive in 34%, with either cytochemical staining (MPO), or/and flow (CD33, CD34, CD13 and CD117), and/or IHC (CD43, CD68 and lysozyme). 55% were positive for CD43; 46% positive for CD117, 36% positive for CD34, and MPO; 27% positive for CD68.

### 3.2 Cytogenetic-molecular findings

84% of subjects had conventional karyotyping. Cytogenetic abnormalities were identified in 44%. 25% had ≥ 2chromosomal abnormalities. Cytogenetics on bone marrow aspirate revealed either a complex in 2 patients (6.5%) and monosomial del-7q in 2 patients. Core-binding factor B (CBFB) gene rearrangement was the most discovered abnormality in 28% of cases, with RUNX1/RUNX1T1 rearrangement in 7% and del -7q identified in 1 patient. Inv16 was identified in 8; isolated inv (16) (P13.1Q22) in 6, and combined with other additional chromosomal abnormalities in 2 patients (trisomy 8 and complex karyotype). Additionally, FISH was performed in 91% of cases. FISH findings were correlated with conventional karyotyping, however, del-7q was not detected by conventional karyotyping. FLT-3 by RT-PCR was mutated in 45%, (FLT-3-ITD 77%). NGS using TruSight™ Myeloid Sequencing Panel was performed for 54%; pathogenic mutations were identified in 9 patients with VAF of 12–55%, and 4 patients had mutations of unknown significance(VUS). NPM1 and DNMT3A mutations found in 1 patient each. There was no P53, or IDH1 or IDH identified in 9 patients tested. Details of chromosomal abnormalities and somatic mutations illustrated in table- 2.

## 4 Treatment

All patients received conventional anthracycline-cytarabine-based chemotherapy on presentation, either isolated eAML or eAML with marrow involvement, and consolidation therapy applied according to the individual risk profile [18]. Patients in CR, were consolidated with 3–4 courses of intermediate to high dose Cytarabine or offered an allotransplant in CR1. Patients with relapsed disease, were re-treated with non-cross resistant chemotherapy combination, followed by allotransplant if achieved CR.

Two patients underwent surgical resection on presentation (1parotid gland and 2 CNS) and 6 received definitive radiation therapy in a dose of 12–24 Gy in 12 fractions as part of the whole treatment plan. One patient received 6Gy to brain before TBI conditioning before allotransplant for concurrent eAMLwith bone marrow involvement. 29 patients received different antharacycline-cytarabine-based chemotherapy combinations during disease course, 9 received two lines of combination chemotherapy and 16 patients received allotransplant consolidation, including 8 patients in CR1, with different conditioning intensity regimens, and 2 for an isolated eAML relapse.

## 5 Definitions of end points, and statistical methodology

We evaluated epidemiological, clinical, histological, genetic and molecular characteristics and long-term outcomes for all patients using descriptive statistics. CR was defined as complete clinical, radiological resolution of the eAML and <5% of blasts in the bone marrow and no blasts in blood. Partial response(PR) was defined as 50% or more reduction of the largest diameters of the tumor, <5% marrow blasts, and absent blasts in blood. Any other response was considered a failure [18]. Survival was calculated from diagnosis of eAML until death, or last follow up. Median relapse free survival (RFS) was calculated from diagnosis to the date of relapse or last follow up. The effect of age, remission status, cytogenetic risk, treatment applied, anatomic location and allotransplant, on OS and RFS was analyzed using Kaplan–Meier method and log-rank test. All reported P-values are two-sided and P-value < 0.05 was determined significant. The study was approved by institutional Review Board.

## 6 Results

32 patients included in the analysis. Patients detailed characteristics are shown in table- 1. Median age at eAML diagnosis, 33.5 y (range; 1-63y). 29 patients were adults and male to female ratio is 2.2:1. 23 were diagnosed with synchronous bone marrow involvement by AML,7 had prior AML(relapsed) and 9 patients had post-transplant relapse (2 isolated extramedullary relapses). AML was the most common associated hematopoietic malignancy (91%), M − 2 FAB subtype was evident in 38%, followed by M4 and M5 28% and 16% respectively. Twenty patients presented with leukocytosis; and 7 had hyperleukocytosis; anemia in 28, as well as thrombocytopenia in 27 patients. Twenty-five had peripheral blood blasts. LDH was elevated in 23 patients. Other associated diseases included 1 prior solid tumor and 3 MDS/MPN patients with eAML occurring 1 and 2 months after initial diagnosis. Two patients had therapy related eAML with extramedullary disease occurring 2 and 3 months after initial diagnosis of MDS and MPN respectively; along with evidence of monosomy 7, and PTPN11, and complex karyotyping in second patient. Third patient had MDS-II with associated jaw eAML. She received radiation to the jaw, followed by bridging azacitidine, and then allotransplant consolidation. She is in complete remission, 4 years post-transplant.

### 6.1 Response and survival outcomes

Complete remission was achieved in 75% of patients and 19% had relapsed refractory disease; 1patient left untreated and the other died shortly after induction due to sepsis. At a median follow up of 43months (range, 8.6–80mo); 38% were in complete remission, 63% relapsed at a median of 16.56 mo (range; 0.7–80mo) and 69% of patients have died. Ten patients (31%) alive and continued in complete remission. 82% of patients had disease relapse, which was the main reason for increased mortality and shorter survival. 2 patients died due to sepsis,1 due to pulmonary aspergillosis and one induction death was identified.

16 patients (50%) received allotransplant consolidation during the course of eAML (8 were in CR1), at a median time of 12 months (range; 10.80–29mo), from the diagnosis of eAML. CR on day-30 was achieved in all transplanted subjects with full donor chimera and 94% continued to be in CR on day-100. 9 patients relapsed post allotransplant (56%) at a median time of 10.4mo(range,3–59.6mo).

The median overall survival (OS) and relapse free survival (RFS) were 18.4 and 18.7 months respectively for the whole cohort, Fig. 1A+1B. Estimated 4-year OS and RFS were 32%(95%CI:16.3–50.5) and 34%(95%CI:17.3–52.6) respectively. OS was significantly better in patients, who attained CR after induction (12 mo vs zero; P = 0.0001), as well as RFS (12mo vs zero; P = 0.0001) compared to patients with relapsed or refractory disease (Fig. 2A+2B). Estimated 4-year OS of 90.0% vs 5.0% and RFS 100% vs 5%. OS also improved in favorable cytogenetic risk group compared with intermediate and poor risk groups (NR vs 17mo vs 19mo; P = 0.25) based on leukemia net 2017 classification [19].

**Fig. 1A:**
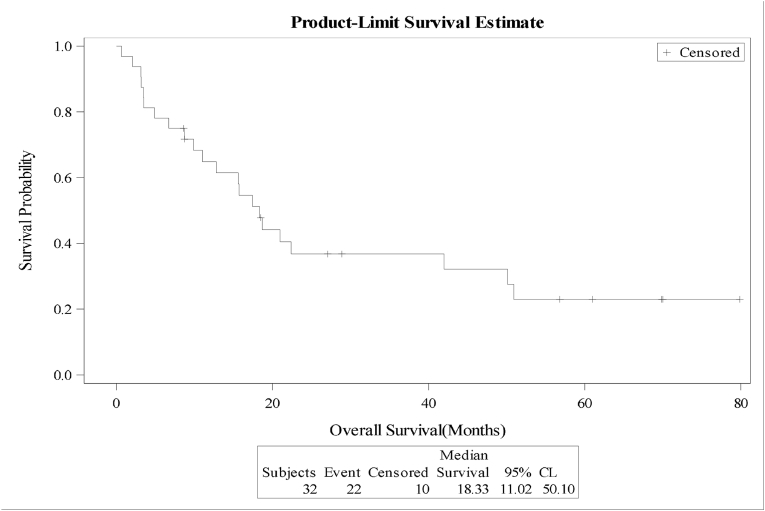
Overall survival (OS) for the entire group(N-32).

**Fig. 1B:**
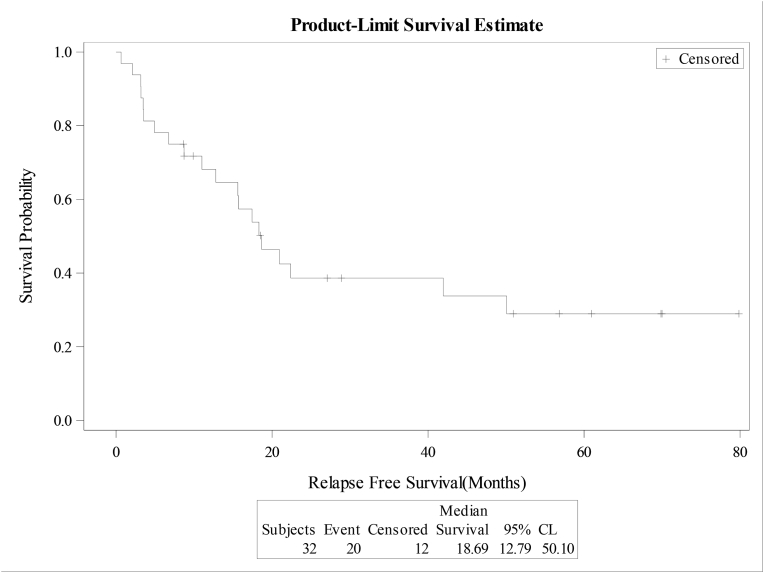
Relapse free survival (RFS) for the entire group(N-32).

**Fig. 2A:**
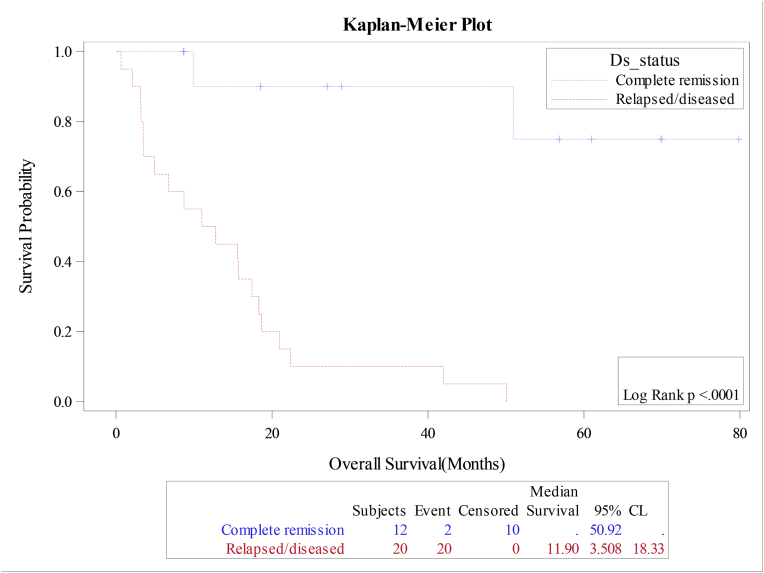
Overall survival according to remission status.

**Fig. 2B:**
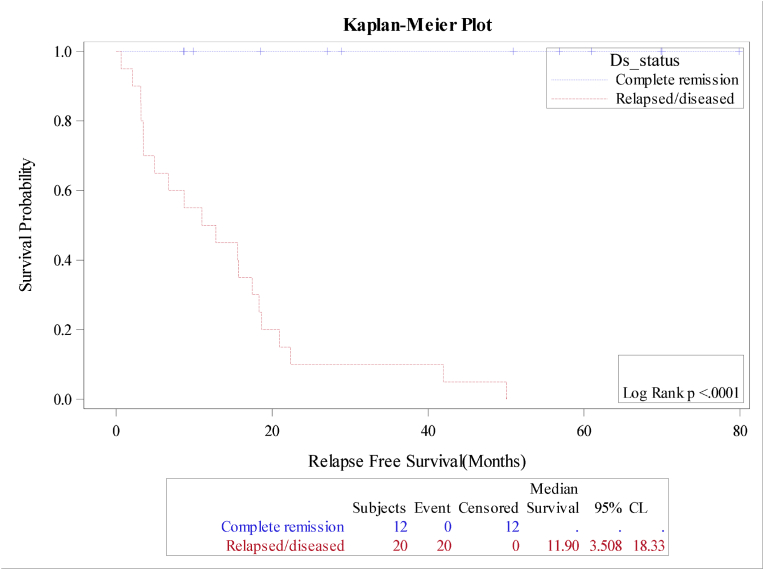
Overall survival according to remission status.

Though, the median OS was improved for patients who received systemic chemotherapy and radiotherapy, the difference was not statistically significant (P = 0.3). However, the OS and RFS were significantly improved in patients, who received allotransplant consolidation with a median OS and RFS of 42mo vs 8mo (P = 0.002) and 42mo vs 10mo (P = 0.006). 2-year OS and RFS 55.0% vs 17%; P = 0.002); and 55.0% vs 20.0; P = 0.006) respectively, Fig. 3A and 3B. Survival outcome did not differ between de-novo eAML and eAMLwith concurrent AML. Additionally, there was no statistical survival outcome correlation with either age (P = 0.668), gender (P = 0.716), location of the tumor (skin versus non-skin, P = 0.818) or LDH serum level (P = 0.05).

**Fig. 3A:**
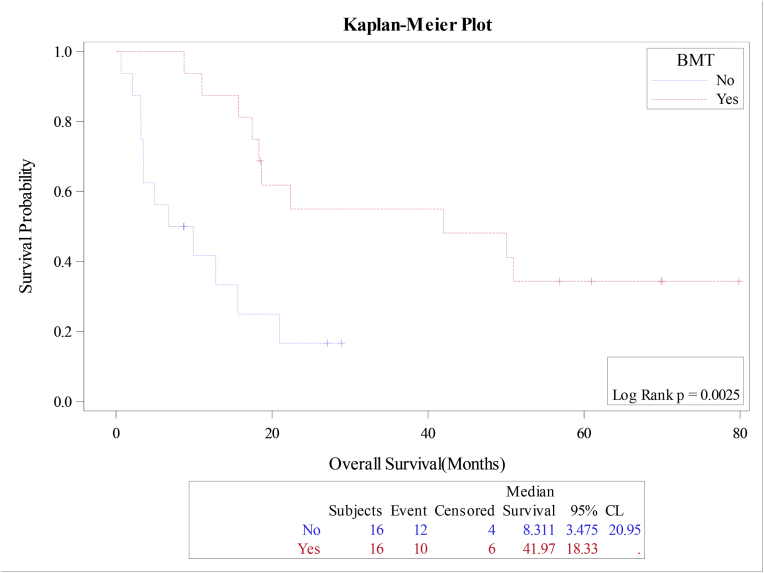
Overall survival (OS) according to bone marrow transplantation(BMT).

**Fig. 3B:**
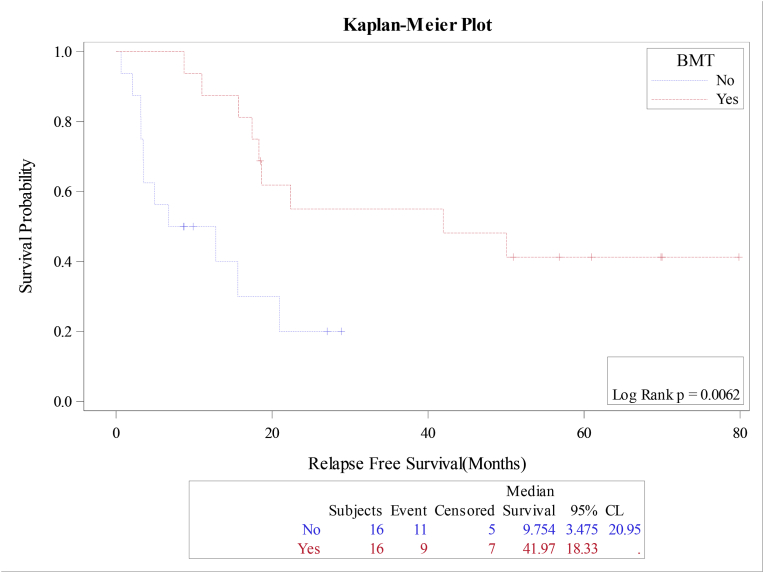
Relapse Free survival (RFS) according to bone marrow transplantation(BMT).

## 7 Discussion

This is the largest study to explore eAML aspects in Jordan. The incidence is 1% during 10 years (N-3147). The majority of patients had AML in the background (63.6%); whereas only 9.4% had MDS/MPN, which is similar to previous large scale reports [[3], [4], [5], [6]]. Though, there was male predominance (68%), the age is younger in our cohort compared to previous reports [4]. Organs most commonly involved was CNS with facial bone invasion (34.4%), lymph-nodes (25%), skin-connective tissue (15.6%), and visceral organs including pancreas, kidneys (25.0%), which is in accordance with previously reported series [5]. Goyal et al. (Blood Cancer J., 2017) reported on 94 000 patients with AML, 0.8% were diagnosed with eAML and the most common sites were connective/soft tissues (31.3%), skin/breast (12.3%), and digestive system (10.3%) [[11], [12], [13], [14]].

The phenotypical profile of our cohort showed similar results, when compared with other reported series but discordant with Al-Khateeb et al., who reported 100% positivity for CD34 [14]. Of note, we observed aberrant expression of CD4 (T-cell markers) in 2 patients. Pileri et al. reported CD68 in 50% of cases compared with 36% in our cohort [4]. All genetic, molecular variables and somatic mutations were collected from bone marrow samples. 40% of patients had normal karyotype and 28% had positive FISH results. Complex and monosomial karyotypes were reported in 4 patients. Chromosomal abnormalities and somatic mutations were detailed in table- 2. We identified rare translocation, like t (7; 12) (q36; p13), t (3; 16) (q10; p10) in 1 patient each. In contrast to the reports previously published on FLT3-ITD and NPM1 mutations occurring in 15% and 28% of patients, we witnessed a higher FLT-3 mutation (45%), and NPM1 identified in 3% of patients [14,20,21]. Al Khateeb et al. reported 16 negative FLT-3 patients out of 21 and Falini et al. had reported the presence of nucleophosmin (NPM) mutation in about 16%, however most of the cases were pediatrics. Seventeen patients had gene sequencing, 76% had pathogenic mutations; 88% had at least one mutation and 23.5% had 2-3 mutations. RTK mutations was higher (56–85%), than reported before (55%) and NRAS is the most common (17.5%), details in table- 2 [[22], [23], [24], [25], [26]].

The outcome patients with eAML is variable and depends on several factors including cytogenetic risk profile, isolated or with concurrent marrow involvement and the presence of any targetable mutations in extramedullary site or marrow samples. The prognosis of eAML and survival outcomes are based on small retrospective series and case reports [6,7,[11], [12], [13], [14]]. Most patients received systemic treatment, 6 patients received chemotherapy and radiotherapy sequentially (19%) and 16 underwent allotransplant consolidation (50%). This is in accordance with the data previously published. Survival outcomes improved with systemic chemotherapy compared with local treatment [6]. Although, high complete remission rate (75.0%) was observed in our cohort, similar to previously reported series, we noted high relapse rate (63%), which comprised 82% of all deaths. This can be explained by high risk cytogenetic profile in our cohort.

Median OS and RFS are reassuring and improved in patients, who went into complete remission after induction (P = 0.0001) and those who received allotransplant (P = 0.03) with corresponding 2-year OS of 55% in allotransplant group compared to 17% for those who did not (P = 0.025). This is comparable with previously reported series [[4], [5], [6]]. Survival outcome did not differ according to disease site with similar 4-year OS 40.0 versus 31.8%(skin-soft tissue vs other). Unfortunately, we cannot draw any conclusion in patients with MDS/MPN in the background because of small sample size, being isolated or with synchronous disease and according to LDH serum level.

In our cohort, 16 patients successfully underwent allotransplant and long-term remissions were achieved in 8 patients, which is similar to EBMT published registry data [16]. Acknowledging the retrospective nature of the study, small number of patients, who receive allotransplant, with reasonable median follow up of 38 months(range,8.6–80mo), we observed long term remissions, which is in accordance with outcomes noted in previous single institute series [4,5,16]. Thus allotransplant may be considered for all eligible patients in first CR [6,16].

This study includes a review of 32 cases of eAML managed at our institute over 10-year period. Our results are fared important, but the study had several limitations. This is a retrospective study with relatively small number of patients. Also, we could not analyze cytogenetic-molecular aberrations and targetable mutations from extramedullary tissue, that may dictate treatment approaches, which may affect long-term survival outcomes. Our work, however, provides detailed clinical, pathological and genetic-molecular information, treatment outcomes. It adds valuable points on treatment approaches, offers hypothesis generating information about the efficacy of allotransplant, and identification of potential prognostic and predictive features such as achievement of CR, cytogenetic risk profile, that may help in risk stratification of patients for future randomized trials in eAML.

## 8 Conclusions

eAML is rare entity, which requires utilization of advanced diagnostic methods including gene sequencing to identify targetable mutations, that may dictate therapeutic options. Remission induction using AML type chemotherapy remains the standard of care. Consolidation therapy remains controversial, and should be indivisialized depending on extent of disease, cytogenetic risk profile, age and performance status. Inclusion of patients with eAML in large prospective clinical studies, is preferred to better identify the best treatment approaches.

## Funding

There is no funding to this manuscript.

## Ethical approval

NA.

## Consent

NA.

## Registration of research studies

1. Name of the registry:
2. Unique Identifying number or registration ID:
3. Hyperlink to your specific registration (must be publicly accessible and will be checked):

## Guarantor

Khalid Halahleh MD.

## Contribution

Dr. Yazan Alhalaseh collected the initial data. Dalia Al-Rimawi wrote the statistical section and data analysis. K.H wrote the concept, design of the study, formal analysis, wrote the initial draft and final manuscript. NK reviewed the samples. All authors edited the manuscript and approved the final draft.

## Ethical approval statement

The study protocol was approved by the institutional review board of King Hussein Cancer Center.

## Data availability statement

The data that support the findings of this study are available from the corresponding author upon reasonable request.

## Provenance and peer review

Not commissioned, externally peer-reviewed.

## Declaration of competing interest

None.
